# Distinct cellular toxicity of two mutant huntingtin mRNA variants due to translation regulation

**DOI:** 10.1371/journal.pone.0177610

**Published:** 2017-05-11

**Authors:** Haifei Xu, Juan Ji An, Baoji Xu

**Affiliations:** Department of Neuroscience, The Scripps Research Institute Florida, Jupiter, Florida, United States of America; Hokkaido Daigaku, JAPAN

## Abstract

Huntington’s disease (HD) is a neurodegenerative disorder caused by CAG repeat expansion within exon1 of the *HTT* gene. The gene generates two mRNA variants that carry either a short or long 3′ untranslated region (3′UTR) while encoding the same protein. It remains unknown whether the two mRNA variants play distinct roles in HD pathogenesis. We found that the long *HTT* 3′UTR was capable of guiding mRNA to neuronal dendrites, suggesting that some long-form *HTT* mRNA is transported to dendrites for local protein synthesis. To assay roles of two *HTT* mRNA variants in cell bodies, we expressed mRNA harboring *HTT* exon1 containing 23x or 145x CAGs with the short or long 3′UTR. We found that mutant mRNA containing the short 3′UTR produced more protein aggregates and caused more apoptosis in both cultured neurons and HEK293 cells, compared with mutant mRNA containing the long 3′UTR. Although the two 3′UTRs did not affect mRNA stability, we detected higher levels of protein synthesis from mRNA containing the short 3′UTR than from mRNA containing the long 3′UTR. These results indicate that the long *HTT* 3′UTR suppresses translation. Thus, short-form mutant *HTT* mRNA will be more efficient in producing toxic protein than its long-form counterpart.

## Introduction

Huntington’s disease (HD), an inherited autosomal dominant neurodegenerative disorder, is characterized by motor disturbance, cognitive loss, and psychiatric manifestations [1]. It is caused by an unstable CAG repeat expansion within exon1 of the *HTT* gene that encodes huntingtin protein [2]. Corresponding to the CAG genetic expansion, there is an abnormally long polyglutamine (polyQ) tract within the N-terminal region of mutant huntingtin (mHtt). The mHtt exhibits toxic properties that lead to dysfunction and death of neurons, primarily in striatal and cortical areas [3]. The N-terminal fragment of mHtt, which is encoded by *HTT* exon1, has attracted much attention because it is generated *in vivo* and can induce HD-like pathology when expressed in mice [4, 5].

The expansion of the polyQ tract in mHtt causes misfolding of mHtt and formation of protein aggregates in neuronal nuclei and neuropils in HD patients [5, 6]. Once the mHtt concentration reaches a specific level, mHtt monomers initiate formation of dimers, trimers, oligomers, and finally large aggregates [7]. Although it remains unclear if mHtt aggregates are harmful or beneficial, the aggregates reflect accumulation of misfolded mHtt [8]. Misfolded mHtt interferes with a wide range of cellular functions by interacting with both nuclear and cytoplasmic proteins [9–12].

Many mammalian genes produce multiple transcripts that differ in the length of 3′ untranslated region (UTR) while encoding the same protein due to alternative polyadenylation [13]. In fact, more than half of all human genes have multiple polyadenylation sites [13, 14]. The relative abundance of mRNA isoforms with alternative 3′UTRs is highly tissue-specific [15, 16]. Long 3′UTRs contain additional regulatory elements that can regulate subcellular location of mRNA [16, 17], mRNA translation [18–20], and subcellular protein localization [21]. Due to the difference in translational location and efficiency, mRNA isoforms with different lengths of 3′UTRs have distinct biological functions although they encode the same protein [16, 21–23]. The rodent and human *HTT* genes also produce two populations of mRNA species, one with a short 3′UTR (0.64 kb) and the other with a long 3′UTR (3.87 kb), and the relative abundance of these two *HTT* mRNA isoforms varies among tissues [24, 25]. It is unknown whether these two *HTT* mRNAs species have distinct roles in the HD pathogenesis.

In this study we provided evidence that the long *HTT* 3′UTR guides mRNA into neuronal dendrites for local protein synthesis. Although the two forms of *HTT* mRNA had similar distribution and stability in cell bodies, we found that the short form of *HTT* mRNA was translated more efficiently than the long form. This led to more protein aggregates and higher apoptosis rate in cells expressing mutant HTT mRNA with the short *HTT* 3′UTR than those with the long *HTT* 3′UTR.

## Materials and methods

### HEK293 cell culture and transfection

HEK293 cells (ATCC) were grown in Dulbecco’s modified Eagle’s medium (DMEM) (Cellgro) supplemented with 10% fetal bovine serum (Hyclone) and 100 U/ml penicillin/streptomycin (Invitrogen) at 37°C and 5% CO_2_. For transfection, 2 μl of Lipofectamine 2000 (Invitrogen) and plasmid DNA (0.4 μg/kb) were added to 100 μl of DMEM media, respectively and incubated at room temperature for 5 minutes. The two parts were then mixed and incubated at room temperature for an additional 20 minutes before the mixture was added onto HEK 293 cells.

### DNA constructs

To detect the subcellular distribution of endogenous *HTT* mRNAs in rat neurons, we cloned a segment of the *HTT* coding sequence for production of riboprobes through PCR amplification of rat cDNAs. The sequences of PCR primers are as follows: 5′-*GATT* GAATTC cactgctggacagattccga-3′ (forward) and 5′*-GATT* CTCGAGactggtatgatgtggtatcacc-3′ (reverse). The italic part was a random sequence, GAATTC was Eco RI restriction site, and CTCGAG was Xho I restriction site.

The GFP constructs for mRNA localization assays were generated by replacing the BGH (bovine growth hormone) 3′UTR of the previously described GFP-BGH construct [16] with either the human short *HTT* 3′UTR (termed “A”) or the human long *HTT* 3′UTR (termed “AB”). Both the short and long *HTT* 3′UTR were amplified from human genomic DNA using the following PCR primers: 5′-gcgccatggtgggagagact-3′ (common forward), 5′-ggccttgcgattcacatacttta-3′ (reverse for short 3′UTR), and 5′-ttggatgtacaatgtttgcagc-3′ (reverse for long 3′UTR).

To generate constructs pExon1Q23-Myc-A, pExon1Q145-Myc-A, pExon1Q23-Myc-A*B, and pExon1Q145-Myc-A*B for expression of Myc-tagged exon1-encoded N-terminal fragment of huntingtin, we digested plasmids pcDNA3.1-Exon1Q23-Myc and pcDNA3.1-Exon1Q145-Myc (Cat #: CH00036 and CH00046, Coriell Institute for Medical Research) with Pme I and then inserted either the short or long human *HTT* 3′ UTR. In pExon1Q23-Myc-A*B and pExon1Q145-Myc-A*B, the first polyadenylation site was mutated from the sequence TTTAAA to GAATTC, such that the two constructs produce only long 3′UTR *HTT* mRNA. For live cell imaging, the Kaede-containing constructs were created by digesting these four Exon1 constructs with Xho I and Age I to replace the Myc-encoding sequence with the Kaede-encoding sequence, which was PCR amplified with pKaede-N1 (A gift from Michael Davidson, Addgene plasmid #54726) [26] as the template using XhoI- and AgeI-flanked primers: 5′-ctcgagatatgagtctgattaaaccagaaatgaag-3′ (forward) and 5′-accggtttacttgacgttgtccggc-3′ (reverse).

To generate lentiviral constructs for expression of Myc-tagged and exon1-encoded huntingtin fragment, we digested the pUltra vector (A gift from Malcolm Moore, Addgene plasmid #24129) [27] with AgeI and XmaI to remove the 0.8-kb GFP sequence, made the linearized vector have blunt ends, and then inserted the blunt-ended fragment of Exon1Q23 (or Q145)-Myc released from pcDNA3.1-Exon1Q23 (or Q145)-Myc with BamHI and PmeI, generating pUltra-Exon1Q23 (or Q145)-Myc. To generate pUltra-Exon1Q23 (or Q145)-Myc-A construct, we digested pUltra-Exon1Q23 (or Q145)-Myc with BamHI and SalI, and then inserted a sequence that encodes the short *HTT* 3′UTR with compatible restriction sites. To add the long *HTT* 3′UTR to pUltra-Exon1Q23 (or Q145)-Myc, we digested the plasmid with EcoRI and SalI and then inserted a sequence that encodes the long *HTT* 3′UTR with compatible restriction sites. Because the first polyadenylation site was mutated from the sequence TTTAAA to GAATTC, which accidently created an EcoRI restriction site, the final constructs lacked the sequence for the short 3′UTR and were termed pUltra-Exon1Q23 (or Q145)-Myc-B.

### Production of lentivirus

Lentivirus was produced using a standard transient calcium phosphate transfection protocol [28]. In brief, 5 x 10^6^ HEK293 cells were plated in each 10 cm dish coated with 100 μg/ml of PDL. When the culture reached 80–90% confluence, cells were transfected with 1.41 μg/kb/dish of a lentiviral construct, 7.34 μg/dish of pMDL (packaging plasmid expressing gag and pol), 3.96 μg/dish of pVSVG (packaging plasmid expressing envelop protein VSVG) and 2.84 μg/dish of pREV (packaging plasmid expressing Rev). Sixteen hours post-transfection, medium was replaced with fresh DMEM supplemented with 2% FBS and 1% glutamate. Then every 24 hours, media containing lentivirus were collected, and viral particles were concentrated by precipitation with 50% polyethylene glycol 6000 following centrifugation. The virus pellet was resuspended in Neural Basal medium, aliquoted, and stored at −80°C. The titer of a viral preparation was determined on HEK293 cells after a serial dilution.

### Cortical neuron culture

Primary cortical neurons, isolated from E18.5 Sprague Dawley rat embryos, were cultured at a density of 2.0×10^5^ cells per well in 12-well plates, according to procedures described previously [16]. Briefly, all embryos were removed after timed pregnant rats were euthanized using CO_2_. Isolated cortices were digested with 20 U/ml of papain in Hank's Balanced Salt Solution (HBSS) at 37°C for 20 min. Dissociated cells were plated onto 15-mm-diameter coverslips coated with poly-D-lysine (100 μg/ml) in DMEM supplemented with 10% FBS and 100 U/ml penicillin/streptomycin at 37°C and 5% CO_2_. Three to four hours after plating, media was replaced with Neurobasal media (Invitrogen) supplemented with 2% B27, 0.5mM L-glutamine, 0.125 mM glutamate, and 1% penicillin-streptomycin. At 5 days *in vitro* (DIV5), lentivirus was added to culture, and neurons were cultured for another 20 days before being fixed for subsequent immunocytochemistry. All animal procedures were approved by the Scripps Florida Institutional Animal Care and Use Committee (protocol # 13–003).

### Immunocytochemistry

Cultured cells were fixed for 20 min with 4% paraformaldehyde/4% sucrose at room temperature. Cells were washed twice with 1x phosphate-buffered saline (PBS) and permeabilized with 0.25% Triton X-100 in PBS solution. After washing three times with 1x PBS, cells were incubated with blocking buffer (PBS containing 5% BSA and 0.1% Triton X-100) for 1h at room temperature. Afterwards, cells were incubated with primary antibodies in blocking buffer. The following primary antibodies were used: mouse anti-Myc (Sigma-Aldrich, Cat # M4439, 1:30000 dilution), rabbit anti-huntingtin (Millipore, Cat # MAB2166, 1:1000 dilution), rabbit anti-caspase3 (Cell Signaling, Cat # 9661, 1:1000 dilution), mouse anti-MAP2 (Sigma-Aldrich, Cat # 9942, 1:500 dilution). Appropriate DyLight conjugated secondary antibodies (Jackson ImmunoResearch Laboratories) were used at a dilution of 1:500. Nuclei were counterstained with DAPI (Invitrogen, Cat # D1306, 10 mg/ml stock solution, 1:10000 dilution). Images were acquired by using the 40X oil immersion objective under a Nikon C2^+^ confocal microscope. Image analysis was performed using NIH ImageJ software.

### Fluorescence in situ hybridization

Fluorescence *in situ* hybridization (FISH) of primary rat cortical neurons and HEK293 cells on coverslips was performed using DIG-labeled riboprobes and the TSA Plus Fluorescein System (PerkinElmer) according to previously described procedures [16]. After hybridization, cells were treated with RNase A at 37°C to remove unannealed probes. Probe concentrations were 500 ng/ml for *HTT* mRNA and 100 ng/ml for *GFP* mRNA.

### RNA isolation and RT-qPCR

HEK293 cells were transfected with pExon1Q145-Myc-A and pExon1Q145-Myc-A*B. At 24h post transfection, total RNA was isolated using TRIzol Reagent (Invitrogen) according to manufacturer’s instruction. To remove any contaminating genomic and plasmid DNA, total RNA was treated with RNase-free DNase for 30 minutes at 37°C, followed by phenol/chloroform extraction. cDNA was synthesized in the presence with oligo(dT) and a reverse primer complementary to short *HTT* 3′UTR using M-MuLV Reverse Transcriptase (NEB, Cat # M0253). Real-time PCR was performed using the Power SYBR Green PCR master mix (Roche, Cat # 04913914001) with specific gene primers, and gene expression was presented relative to 18S rRNA using 2^−ΔΔCt^ method. The sequences of real-time PCR primers are as follows: 5′-GAGGATCTGAATATGCATACCGG-3′ (forward) and 5′-CATAGGGACCAAGCTGGC-3′ (reverse) for the target gene; 5′-ACAGGATTGACAGATTGA-3′ (forward) and 5′-TATCGGAATTAACCAGACA3′ (reverse) for human 18S rRNA.

### Subcellular fractionation and Western blotting

HEK293 cells were lysed on ice for 60 min in lysis buffer containing 10 mM Tris (pH 7.4), 1% Triton X-100, 150 mM NaCl, 10% glycerol, and freshly added protease inhibitors (Roche Complete Protease Mini, Cat # 4693159001) and phosphatase inhibitors (PhosStop pellets, Sigma Aldrich, Cat # 4906845001). Cell lysates were centrifuged at 15,000 × *g* for 20 min at 4°C. Supernatant was collected as the soluble fraction. The pellet was washed 3 times with lysis buffer and centrifuged at 15,000 × *g* for 5 min each at 4°C. The pellet was resuspended in lysis buffer supplemented with 4% SDS, sonicated 3 times, boiled for 30 min, and collected as the insoluble fraction. Protein concentration was determined using Lowry Protein Assay (Bio-Rad). For Western blot analysis, proteins were subjected to 4%–12% bis-tris midi gels (Bio-Rad) and transferred to nitrocellulose membrane. Membrane was blocked with Odyssey Blocking Buffer (Fisher Scientific). The following primary antibodies were used: mouse anti-Myc (Cell Signaling, Cat # 2276, 1:1000 dilution) and mouse anti-beta tubulin (Sigma-Aldrich, Cat # T8328, 1:8000 dilution). Appropriate IRDye infrared secondary antibodies were purchased from LI-COR Biosciences and used at a dilution of 1:10000. Odyssey Infrared Imaging System (LI-COR Biosciences) was used to detect the signal of target proteins.

### Live cell imaging

HEK293 cells were grown on glass-bottomed 35x35mm dishes and transfected with 0.6 μg/kb of pcDNA3.1-Exon1Q23 (or Q145)-Kaede-A or A*B. Twenty-four hours after transfection, time-lapse imaging was performed using a 40X oil immersion objective under a Nikon C2^+^ confocal microscope (Nikon Instruments Inc.) equipped with a stage-top chamber (INUG2A-TIZ, Tokai Hit Co.). The chamber was humidified and maintained at 37°C with 5% CO_2_. To monitor Kaede synthesis, we first photo-converted the existing Kaede by exposing cells to 405 nm laser with 120 gain and 20% laser power (Nikon Intensilight C-HGFI) for 2 minutes. Newly emerging green-form Kaede was then imaged under 488 nm of excitation laser and a 525/50 nm emission filter at 10-min intervals for 8 hours. The intensity of Kaede fluorescence at the plasma was determined using Nikon software analyzer.

### Statistical analysis

Data are reported as means ± SEM. The significance of differences was analyzed using Student’s *t* test or two-way ANOVA with *post-hoc* Bonferroni correction (* *p* < 0.05, ** *p* < 0.01, and *** *p* < 0.001). A *p* value of less than 0.05 was considered statistically significant.

## Results

### Differential subcellular localization of two *HTT* mRNA variants in neurons

**Fig 1:**
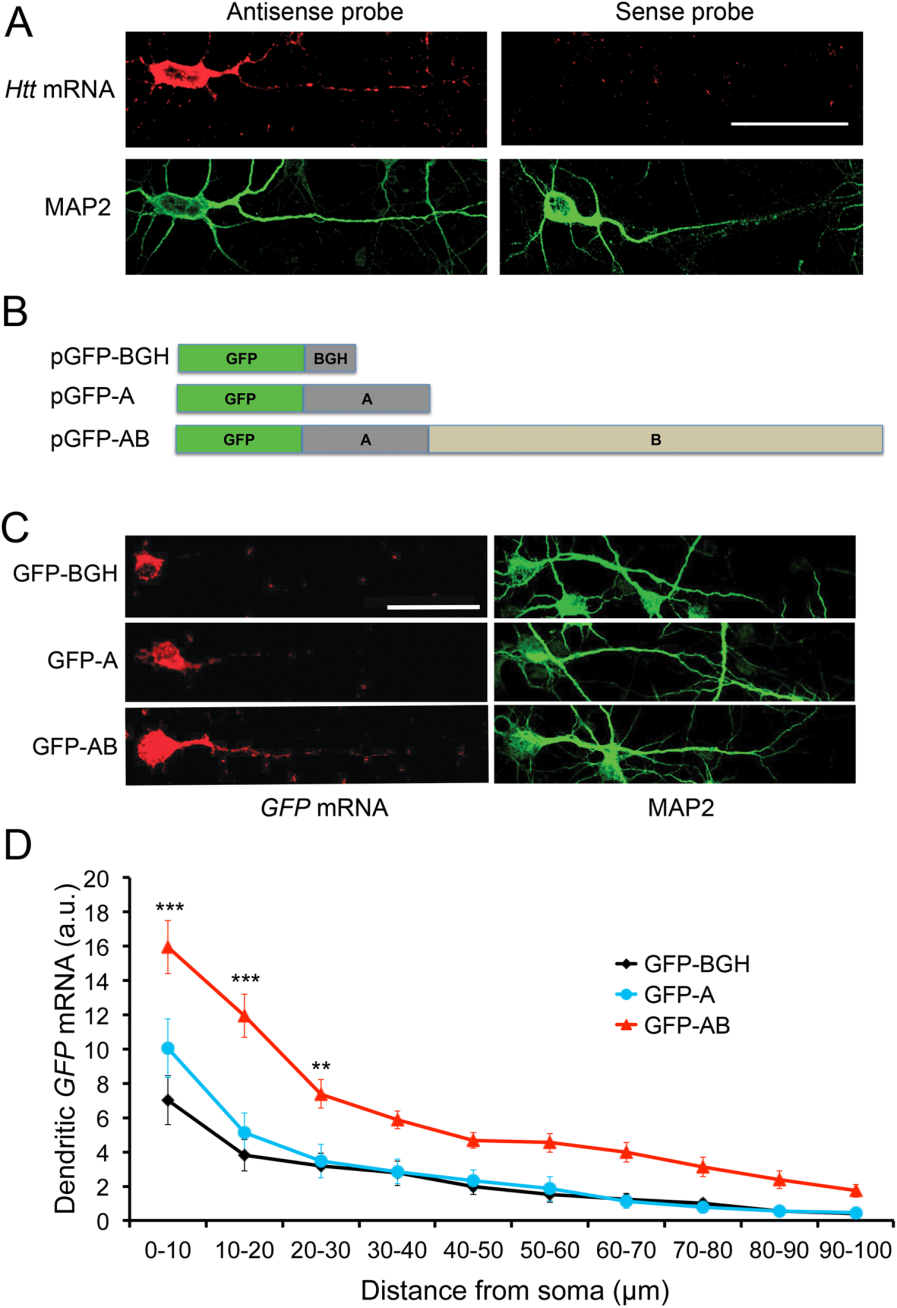
Localization of *HTT* mRNAs in neurons. (A) Distribution of *HTT* mRNA in cell bodies and dendrites of cultured rat cortical neurons. MAP2 immunostaining marks cell bodies and dendrites. The scale bar represents 25 μm. (B) Schematic of three GFP constructs with different 3′UTRs. (C) Representative FISH images of cultured rat cortical neurons transfected with pGFP-BGH, pGFP-A or pGFP-AB. *GFP* mRNA was revealed by FISH using an antisense GFP riboprobe. The scale bar represents 25 μm. (D) Levels of *GFP* mRNAs along main dendrites of transfected neurons. Data are presented in arbitrary units (a.u.). Error bars indicate standard errors. There is a significant difference between the three treatments (*F*_(2, 90)_ = 15.6, *p* < 0.0001; n = 31 neurons per treatment). *Post-hoc* Bonferroni multiple comparisons with the GFP-BGH group: **, *p* < 0.01, ***, *p*<0.001.

Alternative polyadenylation produces two *HTT* mRNA species either with a short or long 3′UTR [24, 25]. Using RNA probes derived from the rat *HTT* coding region, we performed fluorescence in situ hybridization (FISH) on cultured rat cortical neurons. We could detect FISH signals in both cell bodies and distal dendrites (Fig 1A), indicating that some *HTT* mRNA molecules are transported to dendrites. To investigate whether one of the two *HTT* mRNA variants is preferentially transported to dendrites, we cloned the sequences encoding the two *HTT* 3′UTRs from human genomic DNA. We termed the sequence between the stop codon and the first polyadenylation site “A” and the sequence between the first polyadenylation site and the second polyadenylation site “B”. Three constructs were generated to link the GFP-coding sequence with a sequence encoding the 3′UTR for bovine growth hormone (BGH), sequence A (encoding the short *HTT* 3′UTR) or sequence AB (encoding the long *HTT* 3′UTR) (Fig 1B). We transfected the three constructs into cultured rat cortical neurons and detected expressed GFP transcripts using FISH with RNAs derived from the GFP coding sequence as probes. As shown in Fig 1C, GFP transcripts were only detectable in cell bodies and proximal dendrites of neurons transfected with either the GFP-BGH or GFP-A construct. Conversely, GFP transcripts were present in cell bodies and distal dendrites of neurons transfected with the GFP-AB construct. We measured *GFP* mRNA levels along one main dendrite of each transfected neuron. *GFP* mRNA levels in dendrites were significantly higher in neurons expressing GFP-AB than in neurons expressing either GFP-BGH or GFP-A (Fig 1D). There was no significant difference between neurons expressing GFP-BGH and GFP-A in *GFP* mRNA levels along the main dendrite. These results suggest that the vast majority of *HTT* mRNA localized in dendrites should be the long isoform.

### Short 3′UTR *HTT* mRNA produces more mutant huntingtin aggregates than the long 3′UTR isoform in neurons

**Fig 2:**
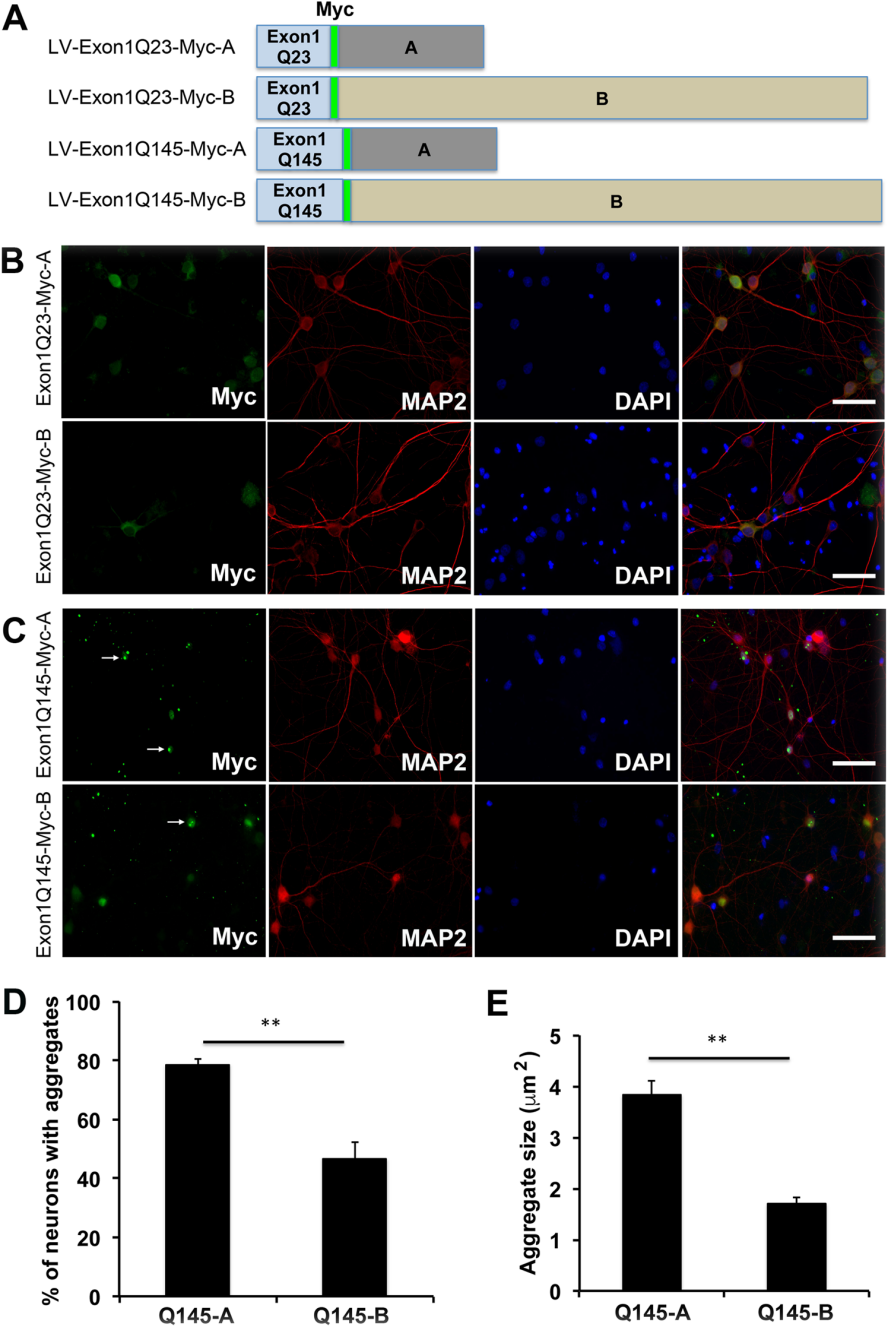
Effects of the *HTT* 3′UTRs on formation of mHtt aggregates in neurons. (A) Diagrams of four lentivirus constructs for expression of a Myc-tagged normal (Q23) or mutant (Q145) Htt N-terminal fragment encoded by Exon1 of the *HTT* gene. The constructs contain genomic sequences encoding the short *HTT* 3′UTR (termed “A”) or the segment of the long *HTT* 3′UTR that lacks the sequence for the short 3′UTR (termed “B”). (B and C) Confocal images of cultured rat cortical neurons, showing Myc-immunoreactive protein aggregates. Neurons were infected with lentivirus at DIV5 and fixed at DIV25 for immunocytochemistry with antibodies against Myc and MAP2. Nuclear DNA was stained with 4′,6-diamidino-2-phenylindole (DAPI). Arrows denote aggregates. Scale bars represent 100 μm. (D) Percentage of neurons containing mHtt aggregates in infected neurons at DIV25 (n = 3 replicates, 340–370 cells analyzed in each replicate). (E) Size of mHTT aggregates in infected neurons at DIV25 (n = 3 replicates, more than 50 aggregates analyzed in each replicate). Error bars indicate standard errors. Student’s *t* test: **p < 0.01.

The two *HTT* 3′UTRs could have impact on mutant huntingtin aggregation by regulating stability, translation, and/or subcellular localization of *HTT* mRNA. To test this hypothesis, we created four lentiviral constructs to express the huntingtin N-terminal fragment in neurons (Fig 2A). The constructs harbor the normal *HTT* Exon1 sequence linked to either the short *HTT* 3′UTR (LV-Exon1Q23-Myc-A) or the sequence unique to the long *HTT* 3′UTR (LV-Exon1Q23-Myc-B), or a mutated *HTT* Exon1 linked to either the short *HTT* 3′UTR (LV-Exon1Q145-Myc-A) or the sequence unique to the long *HTT* 3′UTR (LV-Exon1Q145-Myc-B). The normal Exon1 has a sequence encoding a 23-glutamine tract (Q23), whereas the mutated Exon1 contains a sequence encoding a 145-glutamine tract (Q145). In addition, there is a Myc tag linked to Exon1-encoded huntingtin fragment (Fig 2A).

We infected cultured rat cortical neurons at DIV5 with each of the four lentiviral vectors and then examined Myc immunoreactivity in MAP2-marked neurons at DIV15, DIV20, and DIV25. As revealed by Myc immunoreactivity, no protein aggregates were detected in neurons infected with two lentiviral vectors expressing the normal huntingtin N-terminal fragment (LV-Exon1Q23-Myc-A and LV-Exon1Q23-Myc-B) at any time point (Fig 2B, S1 Fig and S2 Fig). In these neurons the expressed protein was evenly distributed in the soma and dendrite. However, the mutant huntingtin fragment appeared in forms from diffused molecules to aggregates in transduced neurons (Fig 2C, S1 Fig and S2 Fig). At DIV25, many more neurons transduced by LV-Exon1Q145-Myc-A displayed mutant huntingtin aggregates than neurons transduced by LV-Exon1Q145-Myc-B (Fig 2C and 2D). Furthermore, aggregates were significantly larger in neurons transduced by LV-Exon1Q145-Myc-A than in neurons transduced by LV-Exon1Q145-Myc-B (Fig 2C and 2E). These results indicate that the short form of mutant *HTT* mRNA is more efficient in producing mutant huntingtin aggregation in neurons.

### Short 3′UTR *HTT* mRNA also produces more mutant huntingtin aggregates than the long 3′UTR isoform in HEK293 cells

**Fig 3:**
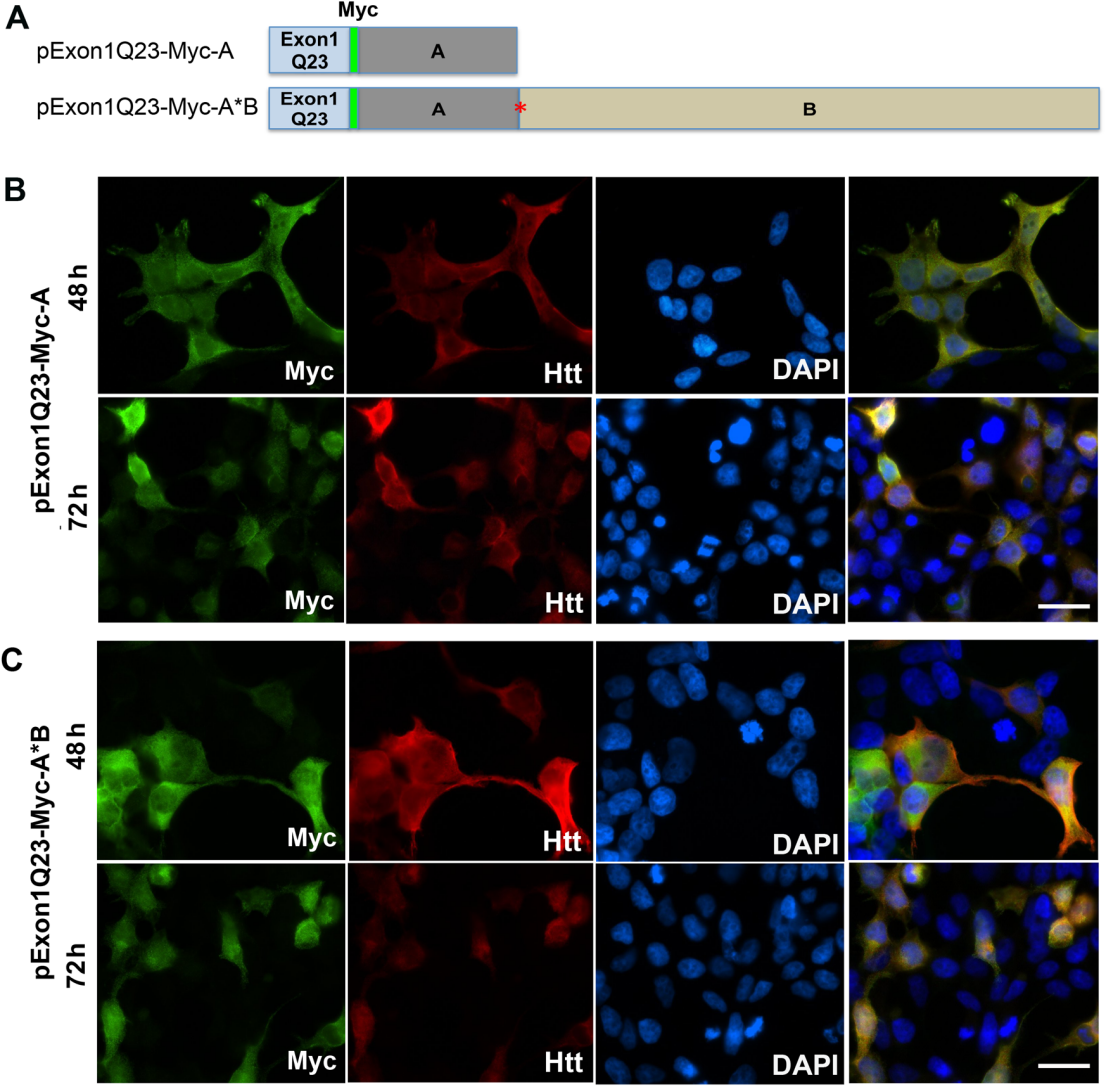
*HTT* 3′UTRs do not affect distribution of normal Htt N-terminal fragment in HEK293 cells. (A) Schematic of normal *HTT* Exon1 constructs with genomic sequences coding for either the short *HTT* 3′UTR (A) or long *HTT* 3′ TR (A*B). Symbol * indicates that first polyadenylation site has been disrupted, such that sequence A*B will only produce the long 3′UTR. Exon1 contains a tract of 23 glutamine-coding codons (Q23). (B) Expression of normal Htt N-terminal fragment from the short 3′UTR construct (pExon1Q23-Myc-A) in HEK293 cells. Transfected cells were stained with antibodies against Myc and huntingtin 48 and 72 h after transfection. Nuclear DNA was stained with DAPI. The experiment was repeated at least 3 times with consistent results. The scale bar represents 100 μm. (C) Expression of normal Htt N-terminal fragment from the long 3′UTR construct (pExon1Q23-Myc-A*B) in HEK293 cells. Transfected cells were stained with antibodies against Myc and huntingtin 48 and 72 h after transfection. Nuclear DNA was stained with DAPI. The experiment was repeated at least 3 times with consistent results. The scale bar represents 100 μm.

**Fig 4:**
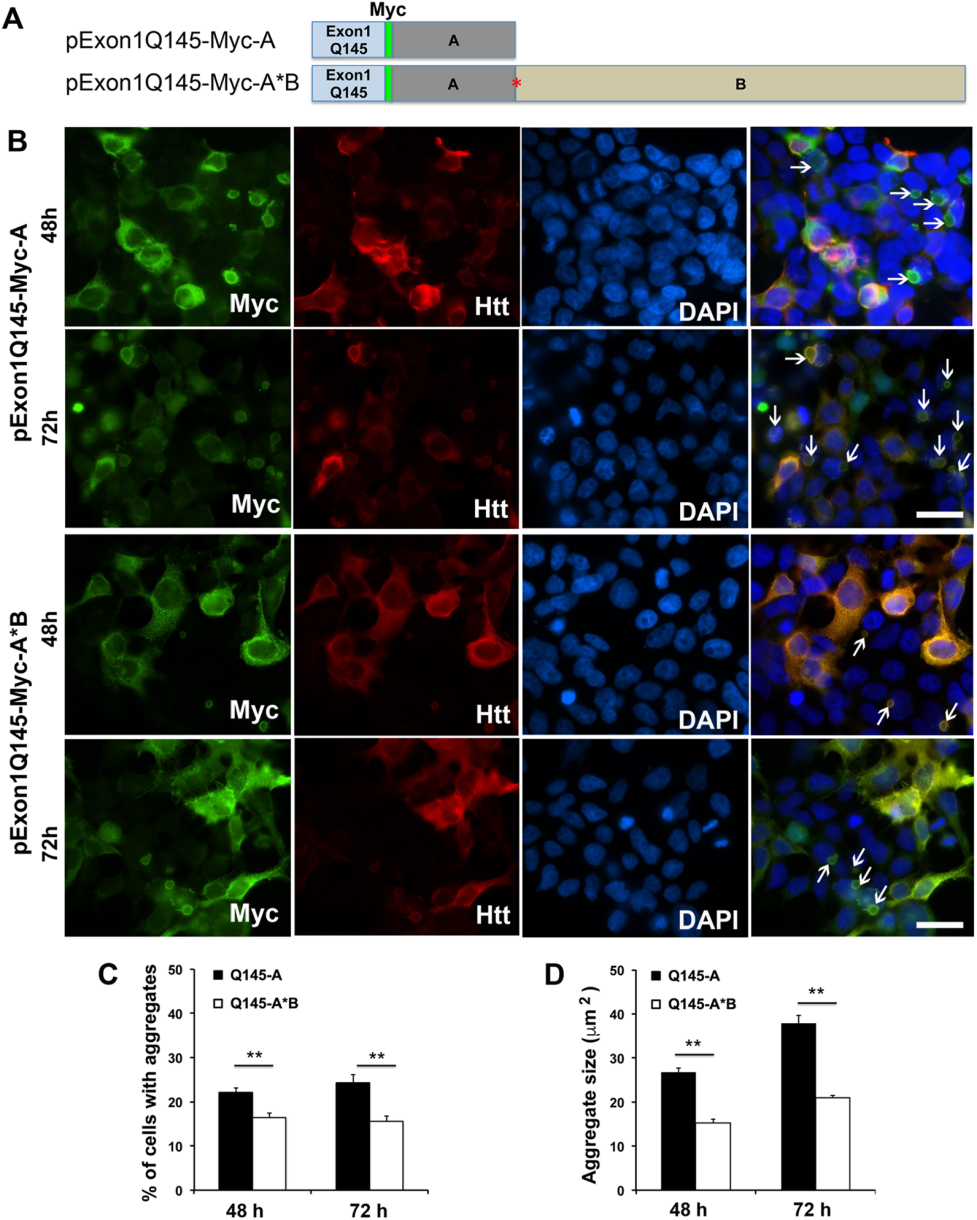
Effects of *HTT* 3′UTRs on formation of protein aggregates in HEK293 cells. (A) Schematic of mutant *HTT* Exon1 constructs. Exon1 contains a tract of 145 glutamine-coding codons (Q145). (B) Expression of mHtt N-terminal fragment from the short 3′UTR construct (pExon1Q145-Myc-A) or the long 3′UTR construct (pExon1Q145-Myc-A*B) in HEK293 cells. Transfected cells were stained with antibodies against Myc and huntingtin 48 and 72 h after transfection. Nuclear DNA was stained with DAPI. Scale bars represent 100 μm. Arrows denote aggregates. (C) Percentage of cells containing mHtt aggregates in transfected cells (n = 4 replicates, 410–700 cells analyzed in each replicate). (D) Size of mHtt aggregates in transfected cells (n = 3 replicates, more than 50 aggregates analyzed in each replicate). Error bars indicate standard errors. Student’s *t* test: **p < 0.01.

One of the reasons why the long form of mutant *HTT* mRNA produces fewer protein aggregates than the short form in neurons could be due to dendritic localization of the long-form *HTT* mRNA and thus its reduced levels in cell bodies. If this is true, the two forms of mutant *HTT* mRNA should produce comparable amounts of protein aggregates in non-neuronal HEK293 cells. To test this hypothesis, we created four mammalian expression constructs to express the N-terminal fragment of huntingtin in HEK293 cells (Figs 3A and 4A). The expression cassettes in these constructs are identical to those in the lentivral constructs except for the sequence encoding the long 3′UTR. The sequence (termed A*B) encodes a full-length long *HTT* 3′UTR and contains mutations at the first polyadenylation site, such that it produces only the long 3′UTR.

We transfected the four expression constructs into HEK293 cells and examined the distribution of the expressed Htt fragment at 48h and 72h after transfection using fluorescent immunocytochemistry with antibodies against Myc and Htt. Although the Htt antibody also recognizes the endogenous Htt protein, similar staining patterns from Myc and Htt antibodies (Fig 3B) indicate that Htt immunoreactivity in HEK293 cells was from the overexpressed Htt fragment. The normal Htt fragment with a 23Q tract expressed in cells transfected with either pExon1Q23-Myc-A or pExonQ23-Myc-A*B did not aggregate even 72 h after transfection (Fig 3B and 3C). In contrast, the mHtt fragment with a 145Q tract translated from mRNA with either the short or long *HTT* 3′UTR aggregated to form cytoplasmic and nuclear inclusions in some transfected cells 48 h and 72 h after transfection (Fig 4B). Inclusions could be found in cells expressing the mHtt fragment even at 24 h after transfection (S3 Fig). As observed in neurons (Fig 2), more cells transfected with pExon1Q145-Myc-A (expressing mRNA with the short *HTT* 3′UTR) had aggregates than those transfected with pExon1Q145-Myc-A*B (expressing mRNA with the long *HTT* 3′UTR) (Fig 4C). Furthermore, aggregates were significantly larger in cells expressing mutant *HTT* mRNA with the short 3′UTR than in cells expressing mutant *HTT* mRNA with the long 3′UTR (Fig 4D). These results show that the short form of mutant *HTT* mRNA is more efficient in producing mHtt aggregation than its long-form counterpart in HEK293 cells. They also indicate that dendritic localization of *HTT* mRNA is not the reason why the long *HTT* 3′UTR reduces mHtt aggregation in neurons.

### Soluble and insoluble mHtt in cells expressing short or long 3′UTR *HTT* mRNA

**Fig 5:**
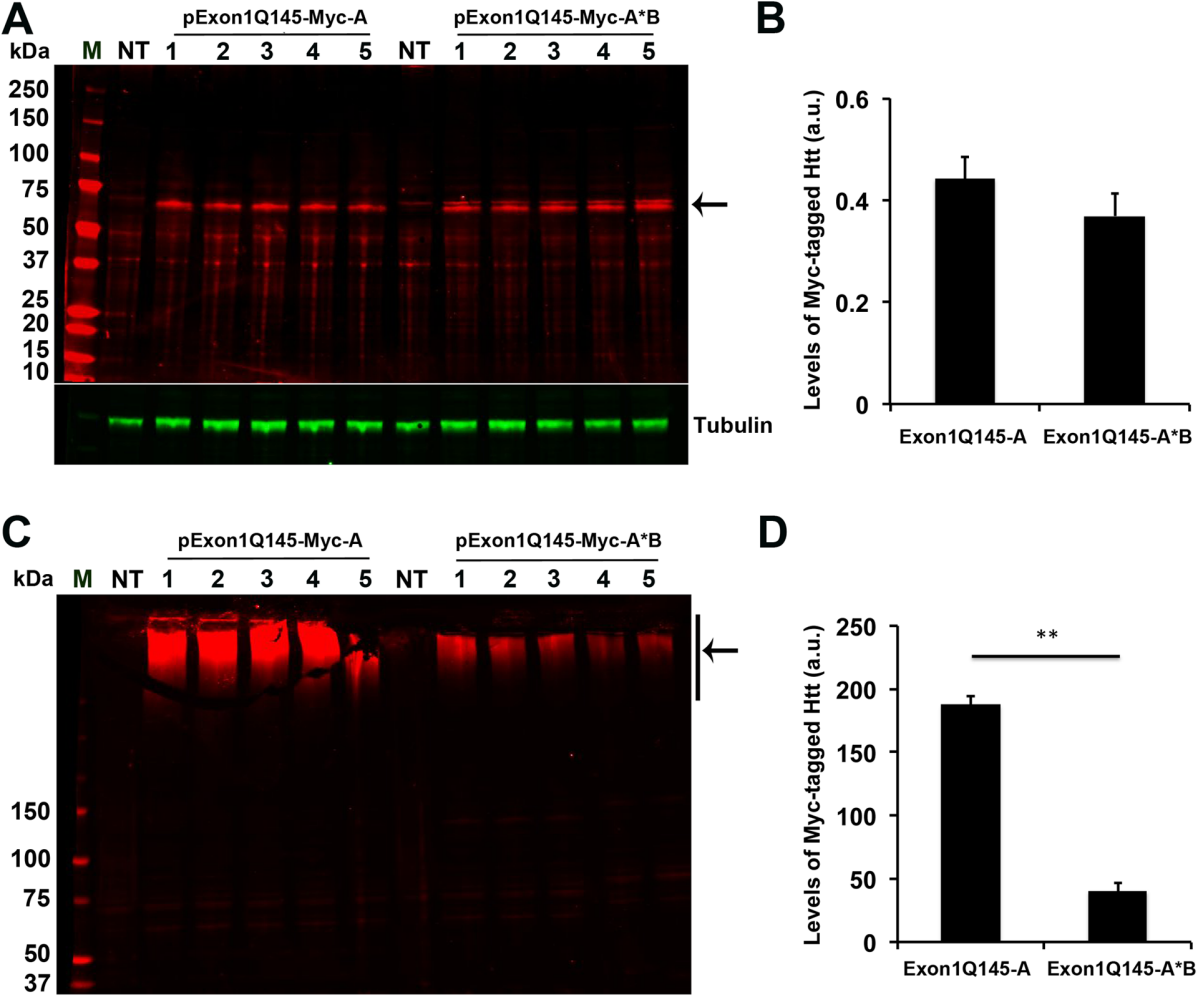
Mutant Htt oligomers in HEK293 cells expressing mRNA with different *HTT* 3′UTRs. (A) Western blot analysis of mHtt in the soluble fraction with an Myc antibody. Cells were transfected with pExon1Q145-Myc-A or pExon1Q145-Myc-A*B and lysed in a buffer containing Triton X-100. M, protein molecular weight size marker. NT, protein extract from non-transfected cells. The arrow indicates expressed mHtt. (B) Quantification of Myc-tagged mHtt levels using densitometry. Levels of mHtt were normalized to amounts of tubulin in the same samples. (C) Western blot analysis of mHtt in the insoluble fraction with an Myc antibody. Cells were transfected with pExon1Q145-Myc-A or pExon1Q145-Myc-A*B and lysed in a buffer containing Triton X-100. Precipitates from centrifugation of lysates (insoluble fraction) were dissolved in an SDS solution. M, protein molecular weight size marker. NT, protein extract from non-transfected cells. The arrow indicates mHtt oligomers. (D) Quantification of Myc-tagged mHtt oligomers using densitometry.

We reasoned that more protein may be produced from short 3′UTR *HTT* mRNA than long 3′UTR *HTT* mRNA, leading to more and larger protein aggregates in cells or neurons expressing mutant *HTT* mRNA with the short 3′UTR than those with the long 3′UTR. Mutant huntingtin is present as both soluble and insoluble protein in cells [29]. We prepared lysates from HEK293 cells transfected with either pExon1Q145-Myc-A or pExon1Q145-Myc-A*B, separated lysates into soluble and insoluble fractions, and analyzed proteins in the two fractions. Immunoblotting analysis with Myc antibodies revealed that the mHtt fragment with a calculated molecular weight of 30 kDa was mainly present as a 60-kDa entity in the soluble fraction (Fig 5A), likely due to formation of SDS-resistant dimers. The levels of soluble mHtt were comparable in the two groups of samples (Fig 5B). Conversely, the insoluble fraction contained high molecular weight of protein species, which are likely oligomers of the mHtt N-terminal fragment (Fig 5C). The level of large mHtt oligomers was 4.7-fold higher in cells transfected with pExon1Q145-Myc-A than those transfected with pExon1Q145-Myc-A*B (Fig 5D). This result indicates that cells expressing short 3′UTR *HTT* mRNA produce much more mHtt protein, the majority of which is present as insoluble oligomers, compared with cells expressing long 3′UTR *HTT* mRNA.

### No effect of the two *HTT* 3′UTRs on levels of *HTT* mRNA in cells

**Fig 6:**
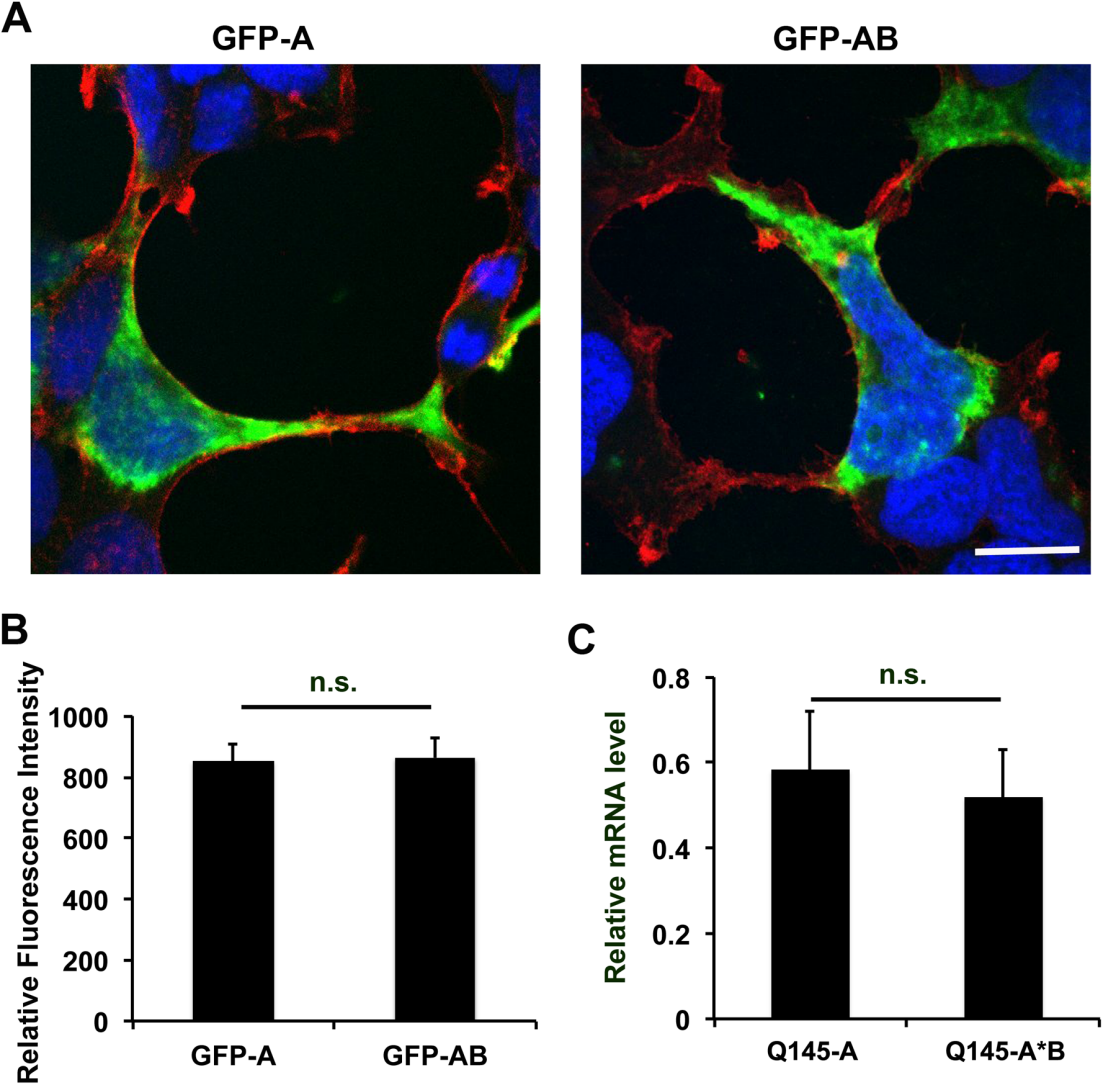
Distribution and stability of mRNA with different *HTT* 3′UTRs. (A) Representative images of FISH, showing distribution of *GFP* mRNA with the short and long *HTT* 3′UTR in HEK293 cells. After GFP FISH (green) was completed, cells were counter stained with phalloidin for actin cytoskeleton (red) and DAPI for nuclei (blue). The scale bar represents 50 μm. (B) Levels of *GFP* mRNA in HEK293 cells. Error bars indicate standard errors (n = 40 cells for each construct). (C) Relative levels of *HTT* exon1 mRNA in HEK293 cells transfected with either pExon1Q145-Myc-A (Q145-A) or pExon1Q145-Myc-A*B (Q145-A*B) (n = 3 for each construct).

Cells transfected with pExon1Q145-Myc-A could have higher levels of Exon1 *HTT* mRNA, which leads to elevated production of mHtt, compared with cells transfected with pExon1Q145-Myc-A*B. We performed FISH to determine levels of *GFP* mRNA in HEK293 cells transfected with either pGFP-A or pGFP-AB (Fig 1B). After FISH, we stained cells with phalloidin to display the cellular cytoskeleton structure. Construct pGFP-AB should produce two *GFP* mRNAs with either the short or long *HTT* 3′UTR. Because long 3′UTR HTT mRNA is more abundant than the short variant in vivo [24, 25], *GFP* mRNA with the long 3′UTR should be the major variant in cells transfected with pGFP-AB. As shown in Fig 6A, *GFP* mRNA showed no obvious difference in subcellular distribution in cells transfected with the two GFP constructs. Furthermore, *GFP* mRNA levels were comparable in cells transfected with the two GFP constructs (Fig 6B). To further examine the effect of the *HTT* 3′UTRs on mRNA levels, we performed real-time RT-PCR to measure *HTT* exon1 mRNA in HEK293 cells transfected with either pExon1Q145-Myc-A or pExon1Q145-Myc-A*B. Again, we found no significant impact of the *HTT* 3′UTRs on mRNA levels (Fig 6C). These results indicate that mRNA abundance is not the reason why cells transfected with pExon1Q145-Myc-A have an elevated level of mHtt oligomers. Because the two GFP constructs have the same promoter and thus transcription should be the same, these results also suggest that the two *HTT* 3′UTRs do not affect mRNA stability.

### Regulation of translation by *HTT* 3′UTRs

**Fig 7:**
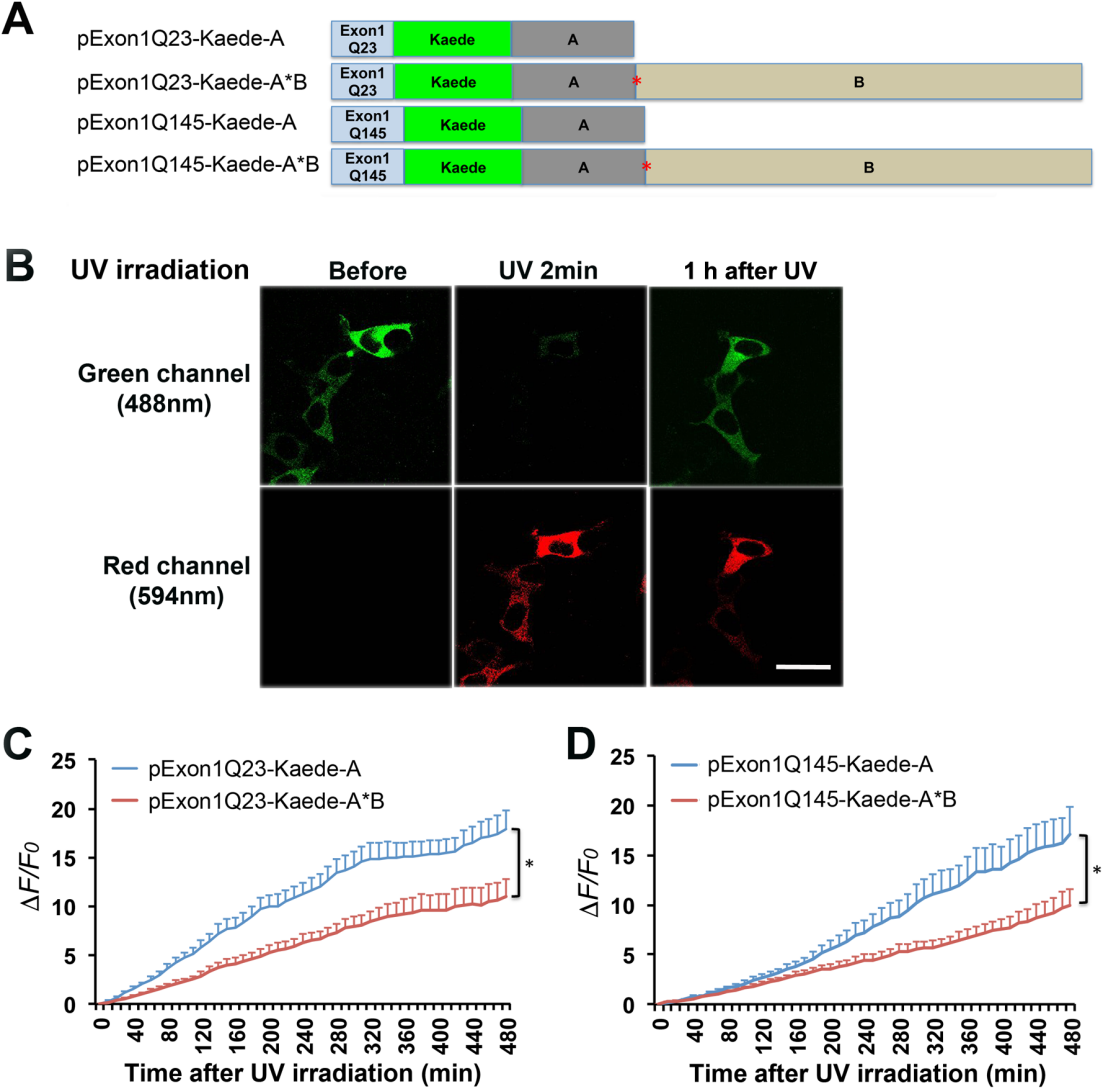
Effects of *HTT* 3′UTRs on protein translation. (A) Schematic of constructs with either the short or long *HTT* 3′UTR for expression of Kaede-tagged normal and mutant HTT N-terminal fragment. (B) Representative images of transfected HEK293 cells, showing photoconversion of Kaede upon UV irradiation. With 2 minutes of UV irradiation, Kaede undergoes irreversible conversion from green fluorescence to red fluorescence. One hour after UV irradiation, newly synthesized Kaede was indicated by the return of green fluorescence. The scale bar represents 100 μm. (C) Levels of newly synthesized Kaede-tagged normal Htt N-terminal fragment in HEK293. Newly synthesized Kaede in a cell was monitored by time-lapse confocal imaging. Its level at time t was quantified as (*F*_t_−*F*_o_)/*F*_o_, where *F*_t_ and *F*_o_ are green fluorescence intensity at time t and immediately after UV irradiation. There is a significant effect from constructs (*F*_(1,102)_ = 6.013, *p* = 0.0159; n = 52 cells for each construct). (D) Levels of newly synthesized Kaede-tagged mHtt N-terminal fragment in HEK293. There is a significant effect from constructs (*F*_(1,99)_ = 6.019, *p* = 0.0159; n = 49 cells for each construct).

We then investigated whether mRNA with the short *HTT* 3′UTR is translated at a higher rate than mRNA with the long *HTT* 3′UTR, which leads to faster accumulation of mHtt. To this end, we employed photoconvertible Kaede protein to examine the effect of two *HTT* 3′UTRs on protein synthesis rate in HEK293 cells. Upon UV light irradiation, Kaede protein is converted from its native green-fluorescence form (Kaede-green) to an irreversible red-fluorescence form (Kaede-red) [30]. We generated four constructs with the short or long *HTT* 3′UTR to express Kaede-tagged normal or mutant Htt N-terminal fragment (Fig 7A).

We transfected HEK293 cells with the four Kaede constructs, irradiated transfected cells with UV light for 2 min, and then monitored green fluorescence from newly synthesized Kaede using time-lapse imaging (Fig 7B). Analysis of fluorescence intensity revealed that Kaede was synthesized at higher rates from the constructs with the short *HTT* 3′UTR than those with the long *HTT* 3′UTR regardless of polyQ tract length in the tagged Htt N-terminal fragment (Fig 7C and 7D and S1–S4 Videos). These results suggest that the additional sequence in the long *HTT* 3′UTR over the short *HTT* 3′UTR (i.e. sequence B) suppresses translation, leading to reduced production of mHtt oligomers in cells transfected with pExon1Q145-Myc-A*B in comparison with those with pExon1Q145-Myc-A.

### Mutant *HTT* mRNA with the short 3′UTR is more toxic than the one with the long 3′UTR

**Fig 8:**
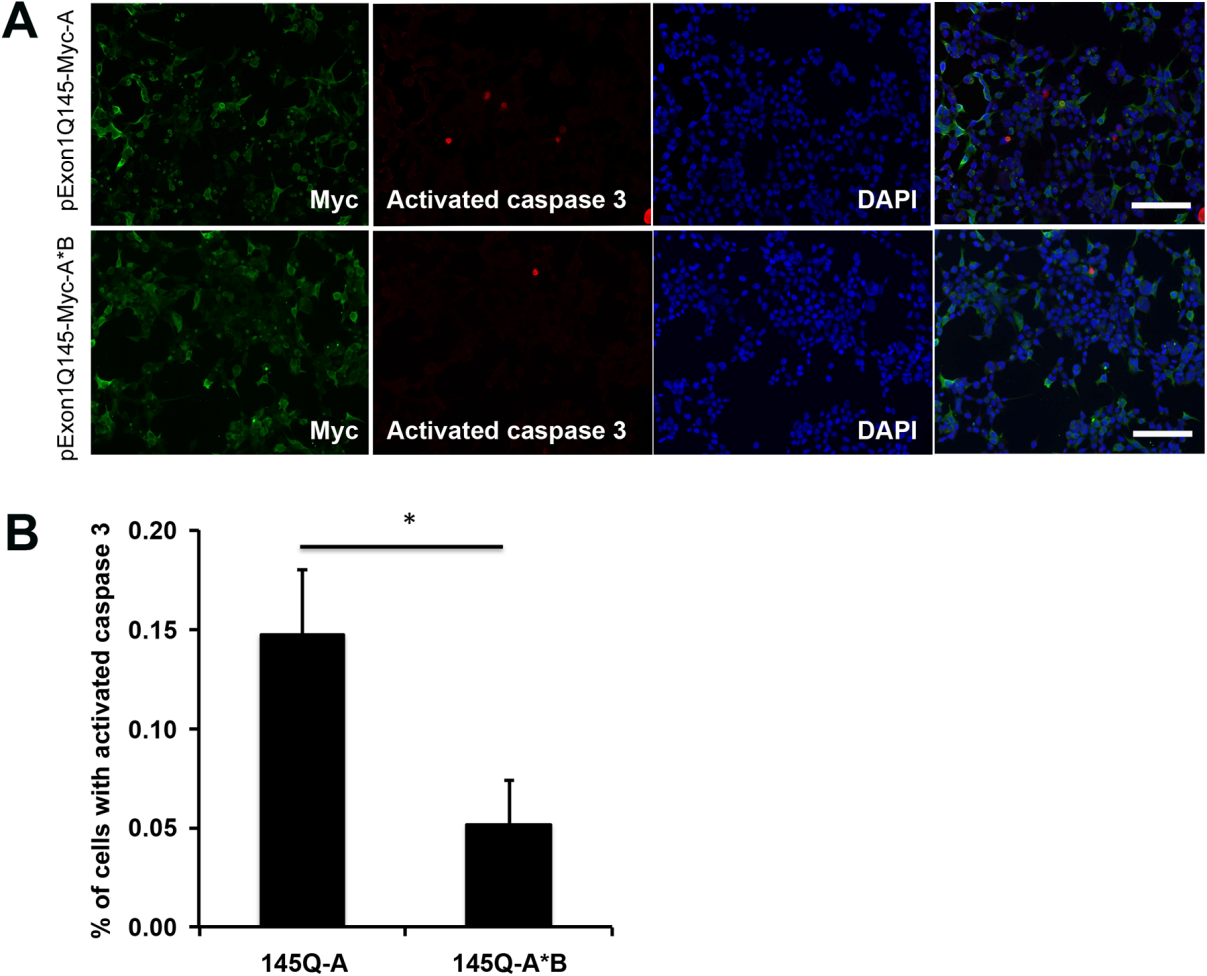
Distinct toxicity of mHtt expressing constructs with different 3′UTRs. (A) Confocal images of HEK293 cells showing apoptosis. HEK293 cells were transfected with pExon1Q145-Myc-A or pExon1Q145-Myc-A*B, stained with antibodies against Myc and activated caspase 3. Scale bars represent 100 μm. (B) Percentage of cells positive for activated caspase 3 over Myc-immunoreactive cells. Student’s *t* test: *p < 0.05.

Mutant *HTT* mRNA with the short 3′UTR is translated at a higher rate and produces more misfolded mHtt, potentially causing more cell death, compared with its counterpart with the long 3′UTR. To test this hypothesis, we transfected HEK293 cells with pExon1Q145-Myc-A or pExon1Q145-Myc-A*B and then detected apoptosis in transfected cells by fluorescence immunocytochemistry using antibodies against Myc and activated caspase 3 (Fig 8A and S4 Fig). Apoptosis rate in cells expressing mHtt from short 3′UTR mRNA was drastically higher than that from long 3′UTR mRNA (Fig 8B). This result indicates that the short variant among the two *HTT* mRNA species present in cells is more toxic when the CAG repeat in the *HTT* gene is expanded.

## Discussion

Similar to many human genes, the *HTT* gene produces two mRNA variants that differ in the length of 3′UTR while producing the same protein due to alternative polyadenylation. Our results indicate that long 3′UTR *HTT* mRNA can be transported to neuronal dendrites for local translation. Furthermore, we found that short 3′UTR *HTT* mRNA is translated at a higher rate than the long isoform in cell bodies, such that cells expressing short 3′UTR mutant *HTT* mRNA display more mHtt aggregates and a higher rate of cell death compared to those expressing the long isoform. These findings suggest that the two isoforms of *HTT* mRNA play distinct roles in the HD pathogenesis.

No studies have been reported to examine dendritic localization of *HTT* mRNA. We were able to detect *HTT* mRNA in dendrites of cultured rat cortical neurons, suggesting that Htt protein can be locally synthesized in dendrites. The observation that the long, but not short, *HTT* 3′UTR is capable of guiding artificial *GFP* mRNA to dendrites of transfected neurons indicates that dendritic *HTT* mRNA should be the long isoform. Among its many cellular roles, Htt is involved in cargo trafficking in neuronal processes [31]. It would be interesting to determine whether normal *HTT* mRNA in dendrites regulates dendritic cargo trafficking and whether mutant *HTT* mRNA in dendrites plays any special role in the HD pathogenesis.

The current study was focused on distinct roles of mutant *HTT* mRNA in cell bodies. We found that mutant *HTT* mRNA with the short 3′UTR produced much more mHtt protein in HEK293 cells, which led to more mHtt aggregates and cell death, than its counterpart with the long 3′UTR, although the two mRNA species had comparable stability. Immunocytochemistry against activated caspase 3 only detected a small percentage of apoptotic cells in the cultures expressing mHtt; however, an apoptotic cell contains activated caspase 3 only for a short period of time. If we assume that caspase 3 is active over a 2-hour window during apoptosis, 0.15% cells positive to activated caspase 3 in the cultures expressing mutant *HTT* mRNA with the short 3′UTR would mean that 1.8% cells die over a day. The number is significant, given that HD takes many years to develop. Because the long *HTT* 3′UTR contains all sequence of the short *HTT* 3′UTR, low translation of long 3′UTR *HTT* mRNA should be due to translation suppression activities of the additional 3′UTR sequence. This explanation is consistent with previous reports that long 3′UTRs suppress translation of *Bdnf* mRNA [20, 32]. Long 3′ UTR *Bdnf* mRNA is largely sequestered into translationally dormant ribonucleoprotein particles at rest, whereas short 3′UTR *Bdnf* mRNA is actively translated to maintain basal production of brain-derived neurotrophic factor (BDNF). Upon neuronal activation, the long *Bdnf* 3′UTR, but not the short *Bdnf* 3′UTR, imparts rapid and robust activation of translation [20]. Similarly, long 3′ UTRs have been reported to negatively affect translation of mRNAs for GluR2, IMP-1, Cyclin D2, and DICER1 [19, 32].

3′UTRs often harbor several binding sites for diverse trans-acting factors, including RNA-binding proteins (RBPs) and microRNAs (miRNAs) [33, 34]. Variation in 3′UTR length can alter translation regulation by inclusion or exclusion of RBP- or miRNA-binding sites. Longer 3′UTR more likely possess such sites, or more of them, and the mRNA will therefore be more likely subjected to negative regulation. Indeed, a systematic examination of 3′UTRs found that 52% of miRNA target sites are located downstream of the first poly A site [35]. Furthermore, it was reported that mRNAs with longer 3′UTRs contained a 2.1-fold higher number of miRNA target sites than those with shorter 3′UTRs in T cells [18]. Consequently, the amount of protein generated by an mRNA is highly related with its 3′UTR length, usually transcripts with shorter 3′UTRs producing higher levels of protein [18]. We speculate that the additional 3.2-kb sequence in the long *HTT* 3′UTRs contains binding sites for miRNAs and/or RBPs that suppress translation. Further investigations are needed to decode the “language” of the *HTT* 3′UTR, such as identification of cis-regulatory elements in the long *HTT* 3′UTR and trans-acting factors. Our findings suggest that the short form of mutant *HTT* mRNA is more toxic than the long form. Reducing production of the short form of mutant *HTT* mRNA by manipulating the polyadenylation process could likely delay the onset of HD.

## Supporting information

S1 Fig Expression of normal and mutant Myc-tagged Htt N-terminal fragment in neurons from constructs with either the short or long *HTT* 3′ UTR. (DOCX)

S2 Fig Expression of normal and mutant Myc-tagged Htt N-terminal fragment in neurons from constructs with either the short or long *HTT* 3′ UTR. (DOCX)

S3 Fig Expression of Myc-tagged Htt N-terminal fragment in HEK293 cells 24 hours after transfection. (DOCX)

S4 Fig Confocal images of HEK293 cells showing apoptosis (red), Myc immunoreactivity (green) and DNA staining (blue). (DOCX)

S1 Video Time-lapse analysis of HEK293 cells transfected with pExon1Q23-Kaede-A. (AVI)

S2 Video Time-lapse analysis of HEK293 cells transfected with pExon1Q23-Kaede-A*B. (AVI)

S3 Video Time-lapse analysis of HEK293 cells transfected with pExon1Q145-Kaede-A. (AVI)

S4 Video Time-lapse analysis of HEK293 cells transfected with pExon1Q145-Kaede-A*B. (AVI)
