# Supplementary material for: Distinct cellular toxicity of two mutant huntingtin mRNA variants due to translation regulation
Source: PLoS One. 2017 May 11;12(5):e0177610. doi: 10.1371/journal.pone.0177610 (PMC5426682; doi:10.1371/journal.pone.0177610)
Supplement: S1 Fig — (DOCX) [file pone.0177610.s001.docx]

**
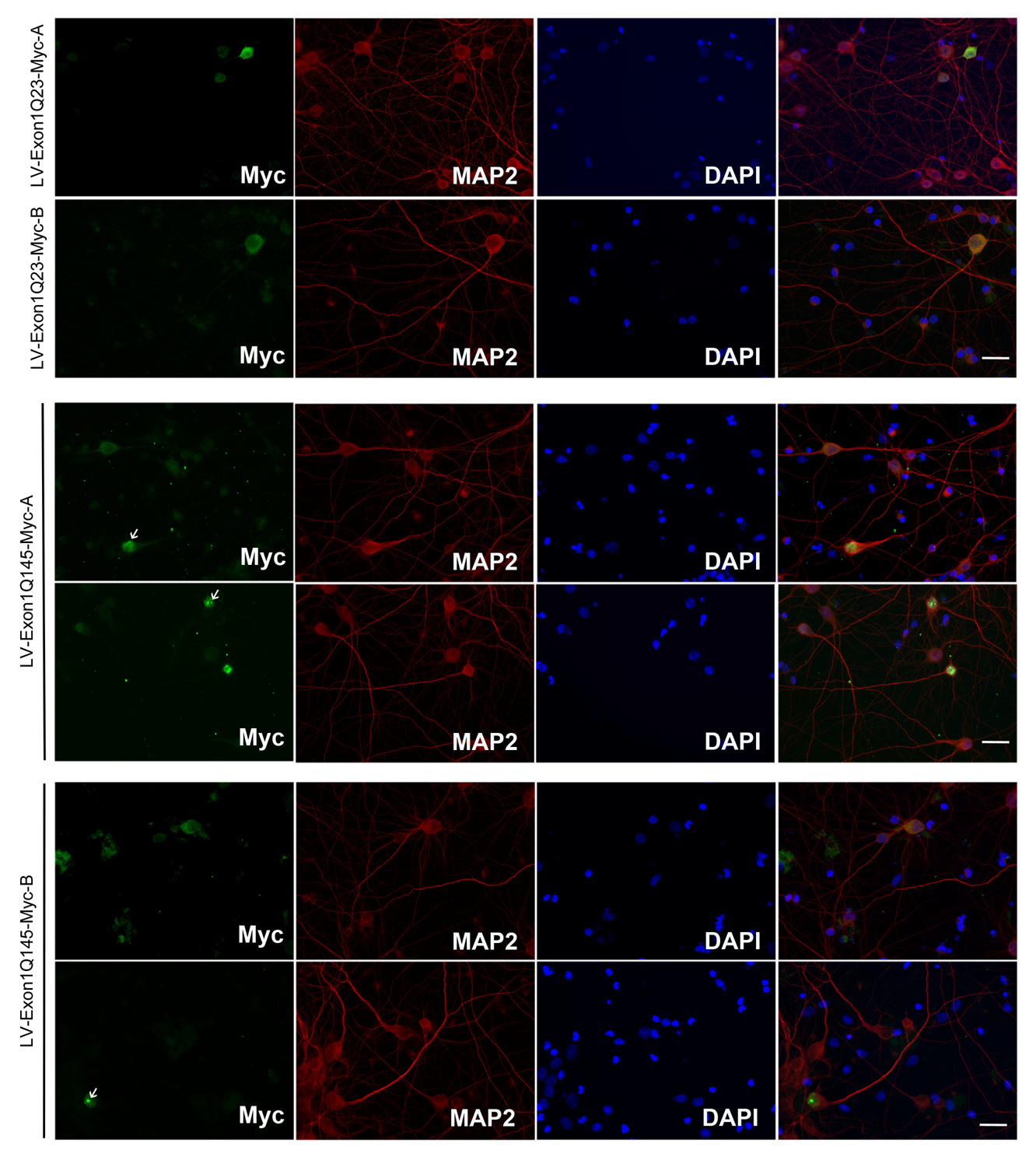
**

**S1 Fig.** **Expression of normal and mutant Myc-tagged Htt N-terminal fragment in neurons from constructs with either the short or long *HTT* 3′ UTR.** Cultured rat cortical neurons were infected with lentiviral vectors at DIV5, fixed at DIV15, and stained with antibodies against Myc and MAP2. Nuclei were stained with DAPI. Arrows indicate protein aggregates. Scale bars represent 100 μm (n=3 individual experiment).
