# Supplementary material for: Distinct cellular toxicity of two mutant huntingtin mRNA variants due to translation regulation
Source: PLoS One. 2017 May 11;12(5):e0177610. doi: 10.1371/journal.pone.0177610 (PMC5426682; doi:10.1371/journal.pone.0177610)
Supplement: S2 Fig — (DOCX) [file pone.0177610.s002.docx]

**
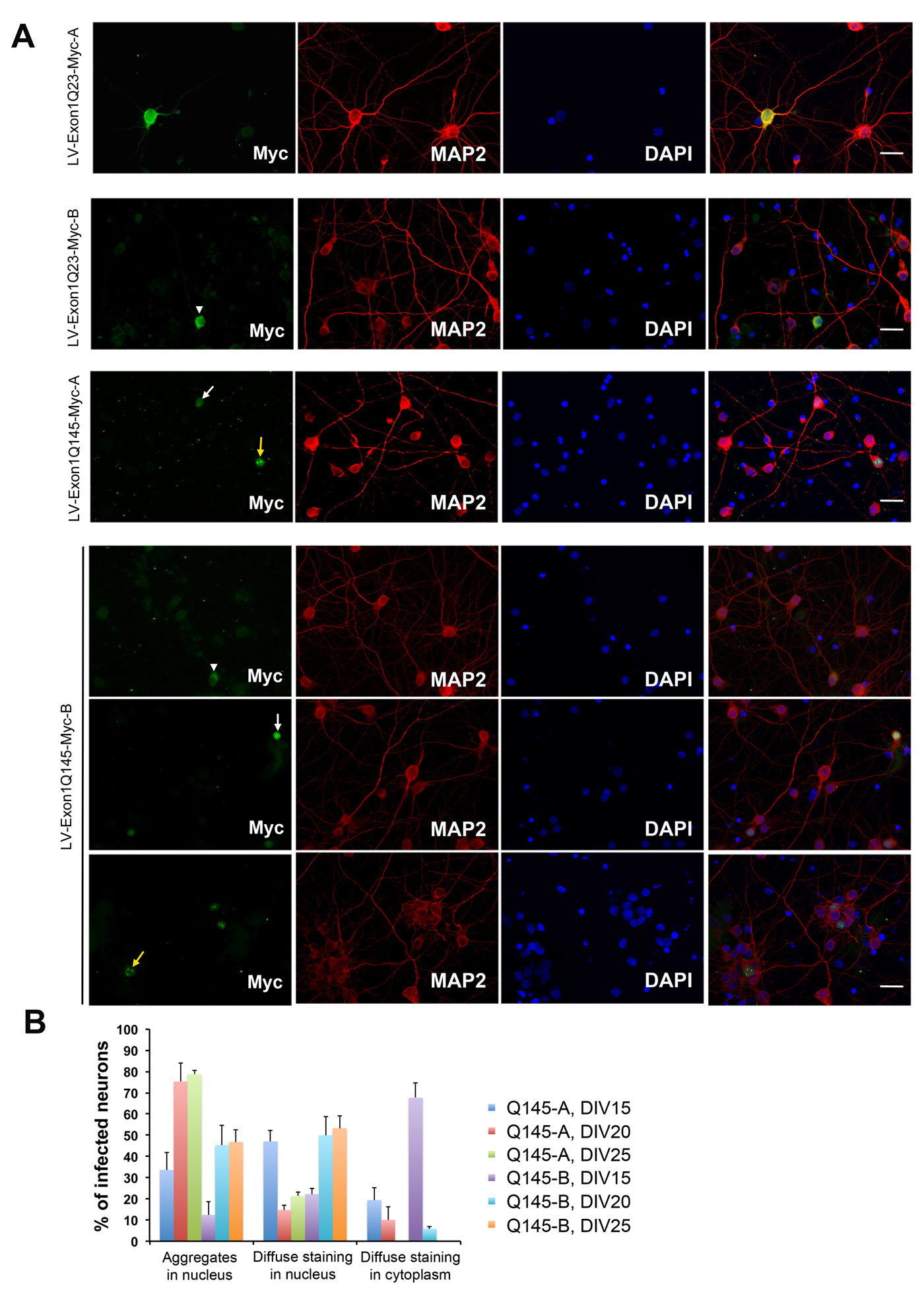
**

**S2 Fig.** **Expression of normal and mutant Myc-tagged Htt N-terminal fragment in neurons from constructs with either the short or long *HTT* 3′ UTR.** (**A**) Immunocytochemistry of cultured rat cortical neurons at DIV20. Neurons were infected with lentiviral vectors at DIV5, fixed at DIV20, and stained with antibodies against Myc and MAP2. Nuclei were stained with DAPI. Arrowheads indicate diffuse staining in the cytoplasm, white arrows diffuse staining in the nucleus, and yellow arrows aggregates in the nucleus. Scale bars represent 100 μm. (**B**) Percentage of infected neurons with diffuse staining in the cytoplasm, diffuse staining in the nucleus, and aggregates in the nucleus (For each condition, 3 individual experiments were repeated).
