# Supplementary material for: Distinct cellular toxicity of two mutant huntingtin mRNA variants due to translation regulation
Source: PLoS One. 2017 May 11;12(5):e0177610. doi: 10.1371/journal.pone.0177610 (PMC5426682; doi:10.1371/journal.pone.0177610)
Supplement: S3 Fig — (DOCX) [file pone.0177610.s003.docx]

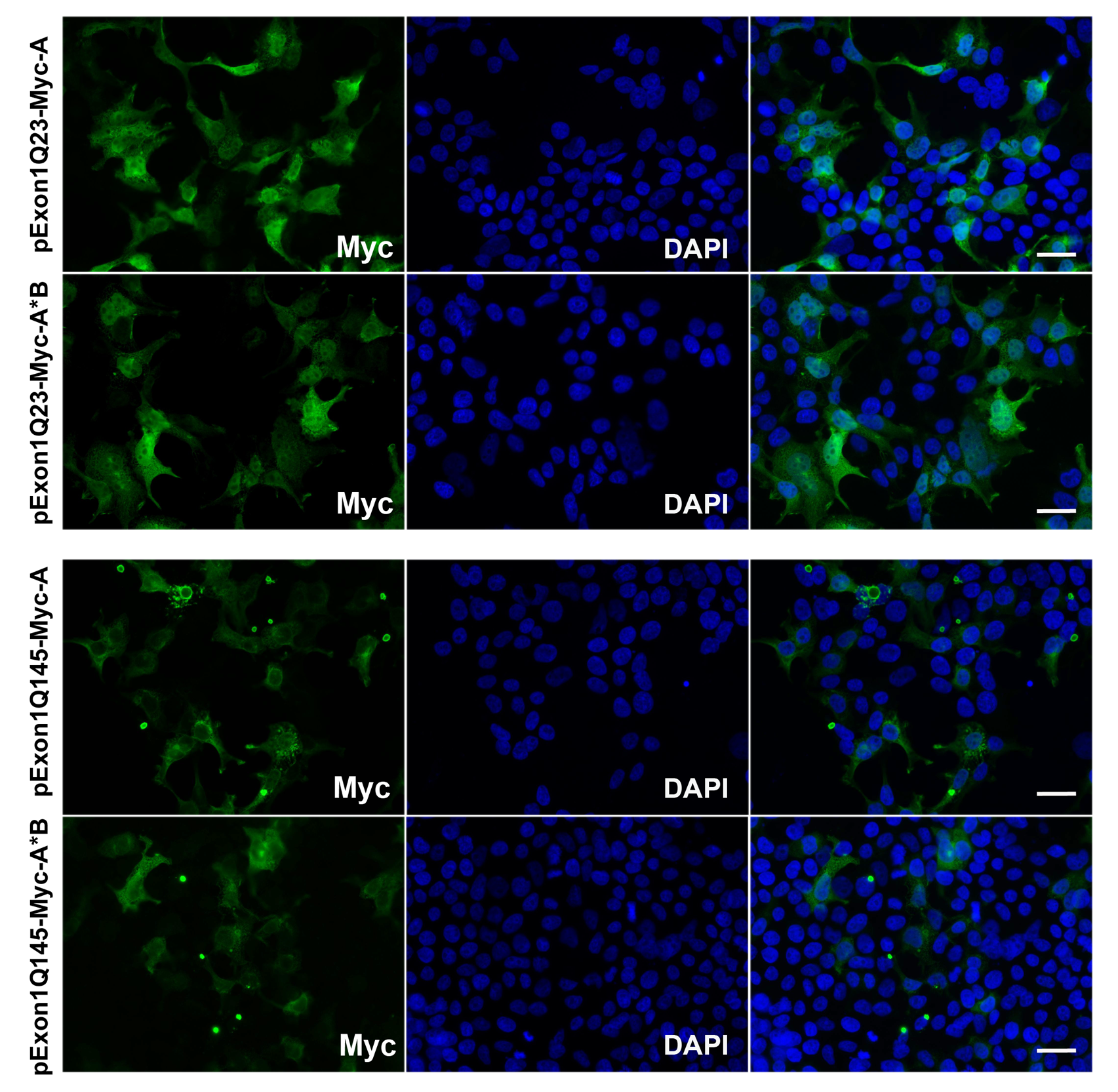


**S3 Fig.** **Expression of Myc-tagged Htt N-terminal fragment in HEK293 cells 24 hours after transfection.** Transfected cells were fixed and stained with antibodies against Myc. Nuclei were stained with DAPI. Scale bars represent 100 μm. For each condition, 3 individual experiments were repeated.
