# Supplementary material for: Distinct cellular toxicity of two mutant huntingtin mRNA variants due to translation regulation
Source: PLoS One. 2017 May 11;12(5):e0177610. doi: 10.1371/journal.pone.0177610 (PMC5426682; doi:10.1371/journal.pone.0177610)
Supplement: S4 Fig — (DOCX) [file pone.0177610.s004.docx]

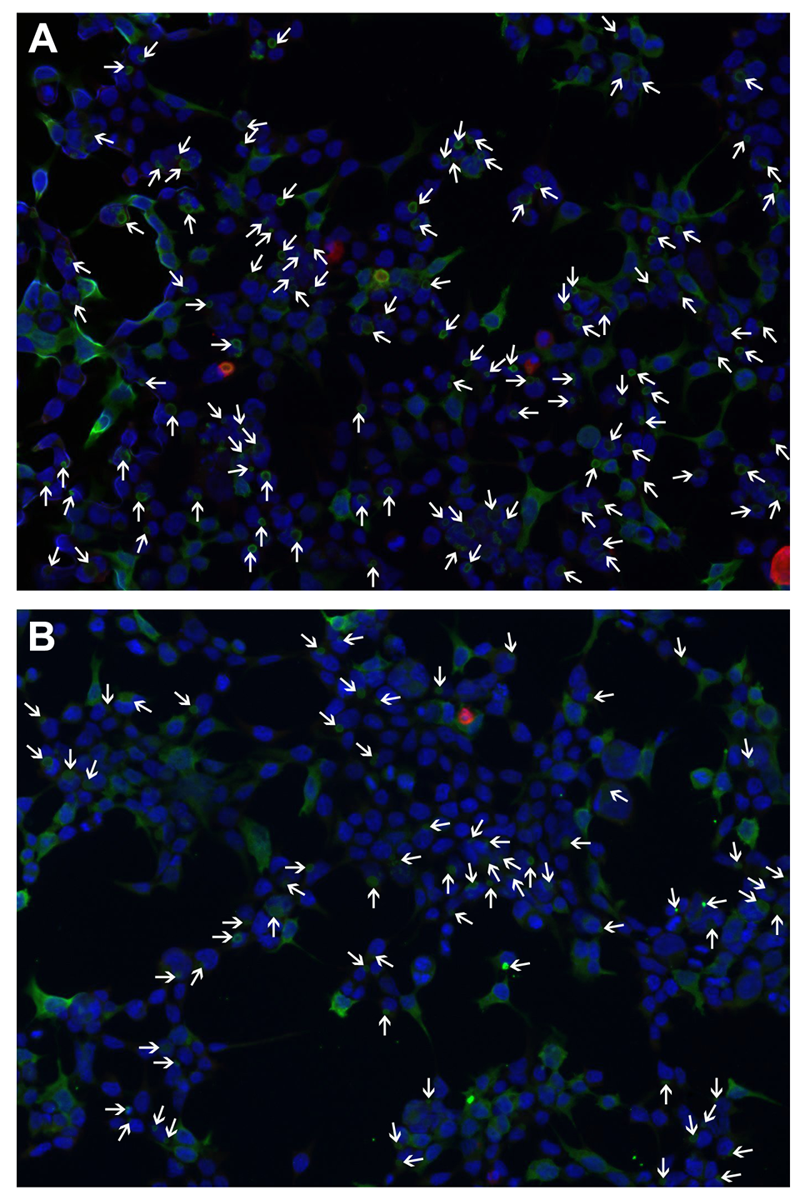


**S4 Fig.** **Confocal images of HEK293 cells showing apoptosis (red), Myc immunoreactivity (green) and DNA staining (blue).** (**A**) HEK293 cells were transfected with pExon1Q145-Myc-A. (**B**) HEK293 cells were transfected with pExon1Q145-Myc-A*B. The images are the enlarged versions of those shown in figure 8A. Arrows indicate Myc-positive protein aggregates.
